# Reduced Levels of Lacrimal Glial Cell Line-Derived Neurotrophic Factor (GDNF) in Patients with Focal Epilepsy and Focal Epilepsy with Comorbid Depression: A Biomarker Candidate

**DOI:** 10.3390/ijms242316818

**Published:** 2023-11-27

**Authors:** Alexander A. Shpak, Flora K. Rider, Tatiana A. Druzhkova, Marina Y. Zhanina, Sofya B. Popova, Alla B. Guekht, Natalia V. Gulyaeva

**Affiliations:** 1 The S. Fyodorov Eye Microsurgery Federal State Institution, 127486 Moscow, Russia; a_shpak@inbox.ru; 2 Moscow Research and Clinical Center for Neuropsychiatry, Moscow Healthcare Department, 115419 Moscow, Russia; danko-2010@yandex.ru (F.K.R.); druzhkova.tatiana@mail.ru (T.A.D.); m.u.kasatkina@gmail.com (M.Y.Z.); sopheternity@mail.ru (S.B.P.); guekht@gmail.com (A.B.G.); 3 Institute of Higher Nervous Activity and Neurophysiology, Russian Academy of Sciences, 117465 Moscow, Russia

**Keywords:** glial cell line-derived neurotrophic factor, brain-derived neurotrophic factor, cortisol, tumor necrosis factor-α, lacrimal fluid, blood serum, focal epilepsy, major depressive disorder, hypothalamic–pituitary–adrenocortical axis

## Abstract

Our previous studies showed that in patients with brain diseases, neurotrophic factors in lacrimal fluid (LF) may change more prominently than in blood serum (BS). Since glial cell line-derived neurotrophic factor (GDNF) is involved in the control of neuronal networks in an epileptic brain, we aimed to assess the GDNF levels in LF and BS as well as the BDNF and the hypothalamic–pituitary–adrenocortical and inflammation indices in BS of patients with focal epilepsy (FE) and epilepsy and comorbid depression (FE + MDD) and to compare them with those of patients with major depressive disorder (MDD) and healthy controls (HC). GDNF levels in BS were similar in patients and HC and higher in FE taking valproates. GDNF levels in LF were significantly lower in all patient groups compared to controls, and independent of drugs used. GDNF concentrations in LF and BS positively correlated in HC, but not in patient groups. BDNF level was lower in BS of patients compared with HC and higher in FE + MDD taking valproates. A reduction in the GDNF level in LF might be an important biomarker of FE. Logistic regression models demonstrated that the probability of FE can be evaluated using GDNF in LF and BDNF in BS; that of MDD using GDNF in LF and cortisol and TNF-α in BS; and that of epilepsy with MDD using GDNF in LF and TNF-α and BDNF in BS.

## 1. Introduction

Epilepsy is a group of non-communicable neurological disorders that affects around 50 million people worldwide. Epilepsy is accompanied by structural and functional changes in neuroplasticity, causing seizures, neurodegeneration, and neural network rearrangement. Neuroplasticity is the basis of brain’s adaptation to changing conditions of the external and internal environment, and the aberrant changes in plasticity cause various neurological and psychiatric diseases related to anxiety, depression, and cognitive dysfunctions, including epilepsy [1]. Increasing number of studies demonstrate that depressive disorders and epilepsy share common mechanisms [2,3] including the dysfunction of the hypothalamic–pituitary–adrenocortical (HPA) axis and neurotrophic factor systems, as well as neuroinflammation [4,5].

Neurotrophic factors are endogenous peptides or small proteins regulating the growth, proliferation, survival, migration, and differentiation of cells in the nervous system and can operate as the potent molecular mediators of the central synaptic plasticity. Most neurotrophic factors can be regarded as neuropeptides that can be synthesized, stored, and secreted by brain cells. Functions of neurotrophic factors are achieved in close interaction with other systems, in particular with the HPA axis and the inflammatory system. The central regulatory role of neurotrophic factors including brain-derived neurotrophic factor (BDNF), ciliary neurotrophic factor (CNTF), glial cell line-derived neurotrophic factor (GDNF), and nerve growth factor (NGF) in neuroplasticity highlights their involvement in the pathogenesis of brain diseases including epilepsy and depression [6,7]. At the ocular surface, neurotrophic factors are involved in the control of immune responses and ocular surface homeostasis in the lacrimal functional unit (conjunctiva, cornea, and tear film) [8]. Though each of these factors has specific functions, they are believed to have neuroprotective and neurorestorative potential [9]. Among other factors, GDNF is supposed to be highly involved in neuronal function and brain diseases, and its use in the diagnostics, prognosis, and treatment of neurological diseases is extensively studied and debated [10,11].

At present, the peptides of the GDNF family and their receptors are regarded as one of the major neurotrophic networks controlling multiple processes in the nervous system [12]. These processes include the development, maintenance, and functioning of both different neurons and glial cells. In a healthy adult brain, GDNF is expressed in neurons, secreted in a paracrine mode, and interacts with neuronal GDNF α1 (GFRα1) receptors. Acting through different signal transduction pathways, GDNF/GFRα1 complex conducts signals to nigro-striatal dopaminergic neurons, motor neurons, enteric neurons, sensory neurons, etc., supporting their survival [13]. However, in an injured brain, GDNF expression occurs in glial cells as well. Importantly, the GDNF expression in both activated astrocytes and microglia is induced by neuroinflammation. Thus, depending on the localization in the brain and the level and duration of glial cell activation, this disease-related GDNF overexpression can be either favorable (potentially adaptive) or harmful. 

Our previous studies showed that neurotrophic factors in the lacrimal fluid (LF) may demonstrate much more pronounced changes than those in BS. Particularly, in patients with focal epilepsy, CNTF levels were increased, while BDNF levels decreased both in the blood serum (BS) and LF, suggesting that high CNTF levels and low BDNF levels in the LF could be considered as non-invasive biomarkers of focal epilepsy [14,15]. Lacrimal GDNF levels were assessed in patients with bipolar disorder and major depressive disease (MDD), and it was shown that a low GDNF concentration in LF could be a potential biomarker of depression [16]. 

Taking into account the potential involvement of GDNF in controlling neuronal networks of epileptic brain [17], the aim of this study was to assess GDNF levels in LF and BS as well as to evaluate HPA and inflammatory indices in patients with focal epilepsy (FE) and in patients with epilepsy and comorbid depression (FE + MDD) compared with patients with depression (MDD) and healthy controls (HC). We also aimed to explore the potential of lacrimal GDNF as a biomarker of epilepsy.

## 2. Results

### 2.1. Characteristics of the Patients and the Healthy Control Groups 

The demographic, clinical, and routine laboratory data of subjects in the FE, FE + MDD, MDD and HC groups, as well as the information about medical treatment and the type and frequency of seizures of the patients are shown in Table 1. The studied groups did not significantly differ in age, gender, education level, and the most routine laboratory data. The hemogram showed few significant differences in the patient groups compared with the HC group (Table 1). Neutrophils were reduced in MDD group, while monocytes were elevated in the FE group, compared to the HC group. FE + MDD and MDD showed augmented levels of lymphocytes (%). MDD had higher prolactin levels compared to HC, most likely due to the higher percentage of patients in the MDD group taking antipsychotics [18].

It should be noted that according to the Beck II scale score, patients with depression (FE + MDD and MDD) differed significantly from patients with FE. In accordance with the MMSE scale score, FE + MDD had a significantly lower cognitive level compared to the MDD group.

### 2.2. Neurotrophic Factors

**Figure 1:**
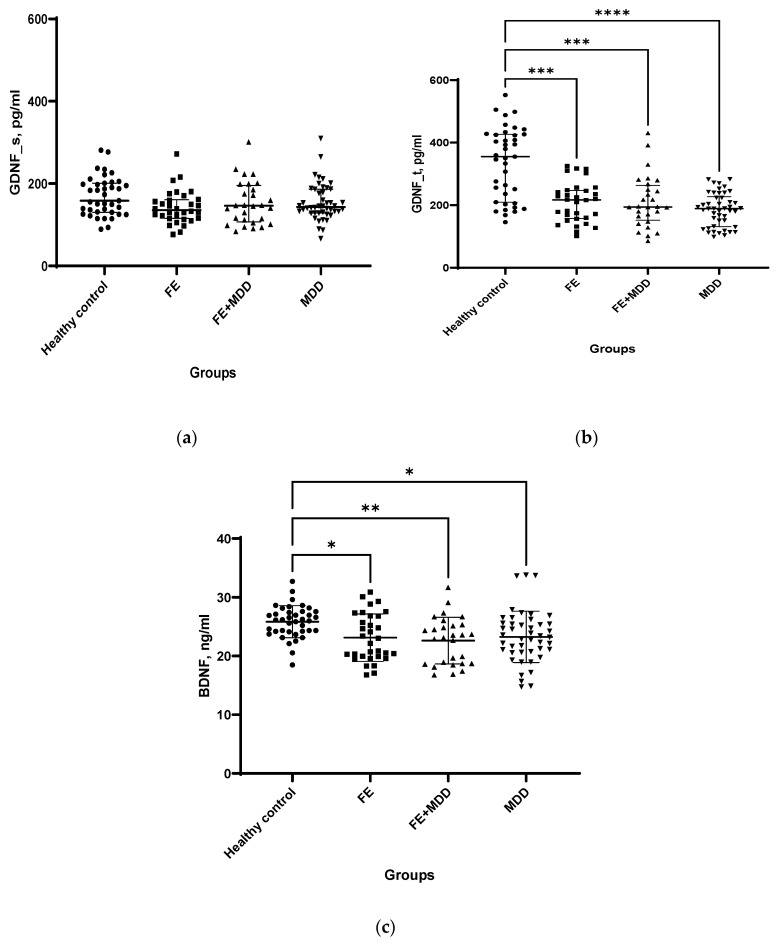
Neurotrophic factors in biological fluids (lacrimal fluid and blood serum) of patients with focal epilepsy (FE), epilepsy and comorbid depression (FE + MDD), and major depressive disorder (MDD) and of healthy controls (HC): GDNF in blood serum (**a**); GDNF in lacrimal fluid (**b**); and BDNF in blood serum (**c**). Kruskal–Wallis test (with post hoc Dunn’s test) for GDNF in BS and GDNF in LF as well as one-way ANOVA (with post hoc Tukey test) for BDNF were used to compare multiple unrelated groups. * *p* < 0.05, ** *p* < 0.01, *** *p* < 0.001, **** *p* < 0.0001.

GDNF levels in BS did not significantly differ between patients and healthy controls (Figure 1a). However, the GDNF concentration in LF was significantly lower in patients compared to the HC group (Figure 1b). BDNF levels were significantly lower in the BS of FE, FE + MDD, and MDD compared to HC (Figure 1c).

### 2.3. Cortisol and TNF-α

**Figure 2:**
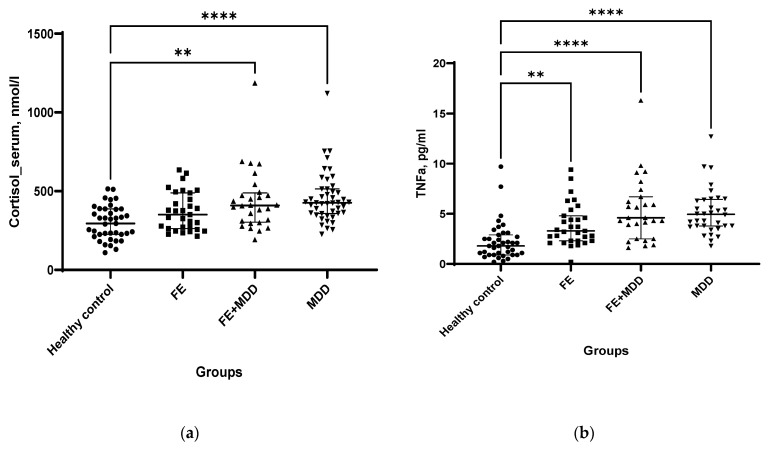
Cortisol (**a**) and TNF-α (**b**) in blood serum of patients with focal epilepsy (FE), epilepsy and comorbid depression (FE + MDD), and major depressive disorder (MDD) and of healthy controls (HC). For the comparisons of unrelated groups, Kruskal–Wallis test adjusted for multiple comparisons and post hoc Dunn’s test were used. ** *p* < 0.01, **** *p* < 0.0001.

Cortisol levels in BS of FE + MDD and MDD were augmented compared with those of the HC group (Figure 2a). When the groups of patients were compared, serum cortisol concentrations were insignificantly higher in MDD than those in FE (trend, *p* < 0.09). The concentrations of TNF-α in BS of all patient groups were increased compared with those of HC (Figure 2b).

### 2.4. Effects of Age, Gender, Medical Treatment, Etiology of Epilepsy, Type and Frequency of Seizures on Lacrimal and Serum GDNF, Serum BDNF, Cortisol, and TNF-α Levels

#### 2.4.1. Age

The levels of lacrimal and serum GDNF, BDNF, cortisol, and TNF-α did not depend on the age of the people participating in the study (Table 2).

#### 2.4.2. Gender

No significant differences were found in lacrimal GDNF, serum GDNF, BDNF, cortisol, and TNF-α levels between men and women (*p* = 0.8, 0.84, 0.72, 0.99, 0.63, respectively). 

#### 2.4.3. Medical Treatment

Serum GDNF levels were higher in FE taking valproates as mono- or polytherapy (median = 148.2 pg/mL) compared with FE not taking medications (median = 115.8 pg/mL) (*p* = 0.005). Other antiepileptic or antidepressant medications did not significantly influence GDNF in BS. GDNF level in LF was not influenced by either of the drugs used.

Serum BDNF levels were higher in FE + MDD taking valproates as mono- or polytherapy (median = 23.71 ng/mL) compared to FE + MDD not taking medications (median = 20.3 ng/mL) (*p* = 0.03).

Serum TNF-α levels were lower in FE not taking medications (median = 4.9 pg/mL) (*p* = 0.03) (median = 3.1 pg/mL) compared with FE taking sodium channel blockers as mono- or polytherapy.

#### 2.4.4. Type or Frequency of Seizures 

Seizure type or seizure frequency did not influence BDNF, cortisol, TNF-α, and GDNF levels in BS and in LF. The comparison of groups with different frequencies of seizures using Kruskal–Wallis test did not reveal any significant effect of the seizure frequency either on scale scores (for MMSE, *p* = 0.14; for Beck II, *p* = 0.17) or the levels of lacrimal or serum GDNF, BDNF, cortisol, and TNF-α (*p* = 0.46; 0.36; 0.81; 0.11; 0.42, respectively).

#### 2.4.5. Etiology of Focal Epilepsy

Levels of lacrimal and serum GDNF, serum BDNF, and cortisol were not significantly different between the groups of patients with focal epilepsy of different etiologies. Serum TNF-α levels were higher in patients with symptomatic focal epilepsy (SFE) associated with traumatic brain injury, stroke, or other cerebrovascular disorders compared to patients with SFE related to other causes (Table 3).

#### 2.4.6. Correlation Analysis

GDNF concentrations in LF and BS showed a significant correlation in only the HC group (R = 0.43; *p*-value 0.01; Spearman rank correlation). In this group, the serum GDNF concentration negatively correlated with the TNF-α level (R = −0.33; *p*-value 0.04; Spearman rank correlation). No significant correlations between LF and BS indices could be found in either group of patients.

### 2.5. Combinations of Biochemical Indices—Potential Predictors for the Probability Assessment of FE, MDD, or FE with MDD

A total of 71 participants were included in the sample (32 patients with FE and 39 healthy volunteers) to generate the model predicting the development of FE, and 85 participants were included in the sample to generate the model to predict the development of MDD (46 patients with MDD and 39 healthy volunteers), and 67 participants (28 patients with FE + MDD and 39 healthy volunteers) were included in the sample to generate the model for predicting the development of FE + MDD. Taking into account the missing values, each of three samples was randomly divided into 80%, assigned to training cohort, and 20%, assigned to internal validation cohort. The following factors were selected as predictors: sex, age, GDNF in LF, GDNF in BS, BDNF, TNF-α, and cortisol. The packages, “caret”, “ordinal”, “visreg”, “yardstick”, and “car”, were used to analyze the data and generate the models.

#### 2.5.1. The Model for FE

**Figure 3:**
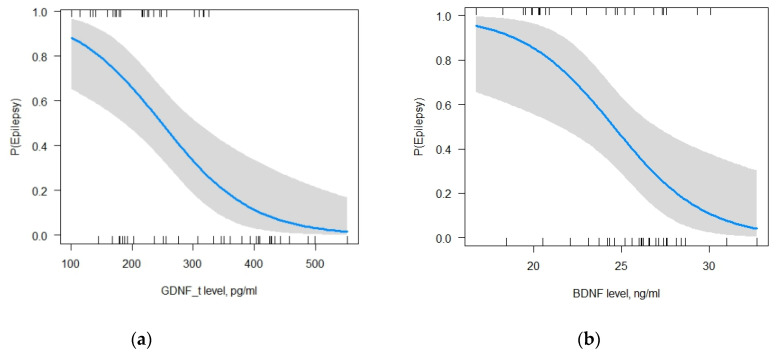
Dependence of changes in GDNF in LF (**a**) and BDNF (**b**) levels on the likelihood of developing epilepsy. Vertical lines along the x-axis indicate observations; gray bar along the blue line indicates a 95% confidence interval.

Multiple logistic regression method was used to estimate the above factors selected as predictors. Only two of them had a statistically significant effect on the occurrence of FE, *p* < 0.05: GDNF in LF (β0 = 10.23, β1 = −0.01, *p* = 0.001; ORβ0 = 7.46*104 (95%CI: 299.8–7.8*107), ORβ1 = 0.98 (95%CI: 0.98–0.99)) and BDNF (β0 = 10.23, β2 = −0.27, *p* = 0.02; ORβ0 = 7.46*104 (95%CI: 299.8–7.8*107), ORβ2 = 0.74 (95%CI: 0.59–0.9)). The increase in the levels of GDNF in LF and BDNF reduces the likelihood of developing FE (Figure 3a,b). 

According to the selected model, reducing the GDNF level in LF by one unit increases the probability of developing FE by 2%, and reducing the BDNF level in BS by one unit increases the probability of FE by 26% (Change in Odds%: (OR-1)*100)). Test for multicollinearity showed that the values of all predictors were close to one. It means that there was no correlation between the explanatory variables of the model (GDNF_t = 1.15, BDNF = 1.15). The AUC of the selected model was 0.85 (accuracy 0.93, precision 1, sensitivity 0.9, f1-score 0.95). Pseudo R-squared values indicate that the model explains 31.4–47% of the data (Pseudo.R.squared/McFadden = 0.31, Cox and Snell = 0.35, Nagelkerke (Cragg and Uhler) = 0.47). The likelihood ratio test is 24.69 with a *p*-value of less than 0.05 that points to the good predictive power of the selected model. After compiling a classification table with the test data of 14 participants (20% of the entire sample) obtained from this model with a cut-off point of 0.5, the precision (how good our model is when the prediction is positive) of the model is found to be 100%, the recall (how good our model is at correctly predicting positive classes) is found to be 90%, and F1 score (integrates precision and recall into a single metric to gain a better understanding of model performance) of the model is found to be 95%. The accuracy of the selected model, with a cut-off point of 0.5 for the binary classification, is 85.7% (a cut-off point of 0.68 is required for 100% accuracy).

#### 2.5.2. The Model for MDD

**Figure 4:**
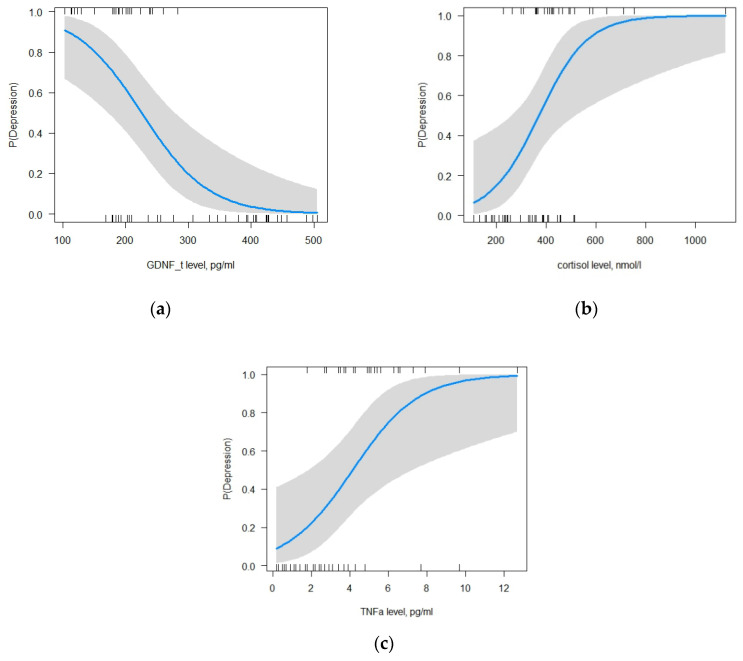
Dependence of changes in GDNF in LF (**a**), cortisol (**b**) and TNF-α (**c**) levels on the likelihood of developing depression. Vertical lines along the x-axis indicate observations; gray bar along the blue line indicates a 95% confidence interval.

For MDD, the multiple logistic regression method showed that out of the seven tested variables, GDNF in LF, cortisol, and TNF-α had a statistically significant effect on the occurrence of MDD, *p* < 0.05: GDNF in LF (β0 = −3.83, β1 = −0.02, *p* = 0.01; ORβ0 = 0.02 (95%CI: 3*10-5-5), ORβ1 = 0.98 (95%CI: 0.97–0.99)); cortisol (β0 = −3.83, β2 = 0.02, *p* = 0.01; ORβ0 = 0.02 (95%CI: 3*10-5-5), ORβ2 = 1.02 (95%CI: 1–1.03)); and TNF-α (β0 = −3.83, β3 = 0.56, *p* = 0.01; ORβ0 = 0.02 (95%CI: 3*10-5-5), ORβ3 = 1.8 (95%CI: 1.22–3.12)). The increase in the level of GDNF in LF and the decrease in the levels of cortisol and TNF-α reduce the likelihood of developing MDD (Figure 4a–c). 

According to the selected model, reducing the GDNF level in LF by one unit increases the probability of developing MDD by 2%, increasing the cortisol level in BS by one unit elevates the probability of developing MDD by 1%, and increasing the TNF-α level by one unit elevates the probability of developing MDD by 80% (Change in Odds%: (OR-1)*100). Test for multicollinearity showed that the values of all predictors were close to one. It means that there was no correlation between the explanatory variables of the model (GDNF in LF = 1.03, cortisol = 1.2, TNF- = 1.18). The AUC of the selected model was one (accuracy 0.93, precision 0.86, sensitivity 1, f1-score 0.92). Pseudo R-squared values indicate that the model explains 64.7–78.8% of the data (Pseudo.R.squared/McFadden = 0.65, Cox and Snell = 0.59, Nagelkerke (Cragg and Uhler) = 0.79). The likelihood ratio test is 51.27 with a *p*-value of less than 0.05 that points to the good predictive power of the selected model. According to the selected model, reducing the GDNF level in LF by one unit increases the probability of developing MDD by 2%, increasing the cortisol level in BS by one unit elevates the probability of developing MDD by 1%, and increasing the TNF-α level by one unit elevates the probability of developing MDD by 80% (Change in Odds%: (OR-1)*100). Test for multicollinearity showed that the values of all predictors were close to one. It means that there was no correlation between the explanatory variables of the model (GDNF in LF = 1.03, cortisol = 1.2, TNF- = 1.18). The AUC of the selected model was one (accuracy 0.93, precision 0.86, sensitivity 1, f1-score 0.92). Pseudo R-squared values indicate that the model explains 64.7–78.8% of the data (Pseudo.R.squared/McFadden = 0.65, Cox and Snell = 0.59, Nagelkerke (Cragg and Uhler) = 0.79). The likelihood ratio test is 51.27 with a *p*-value of less than 0.05 that points to the good predictive power of the selected model. After compiling a classification table with the test data of 15 participants (20% of the entire sample) obtained from this model with a cut-off point of 0.5, the precision of the model is found to be 85.7%, the recall is found to be 100%, and the F1 score of the model is found to be 92.3%. The accuracy of the selected model, with a cut-off point of 0.5 for the binary classification, is 93.3% (a cut-off point of 0.35 is required for 100% accuracy).

#### 2.5.3. The Model for Focal Epilepsy with MDD

**Figure 5:**
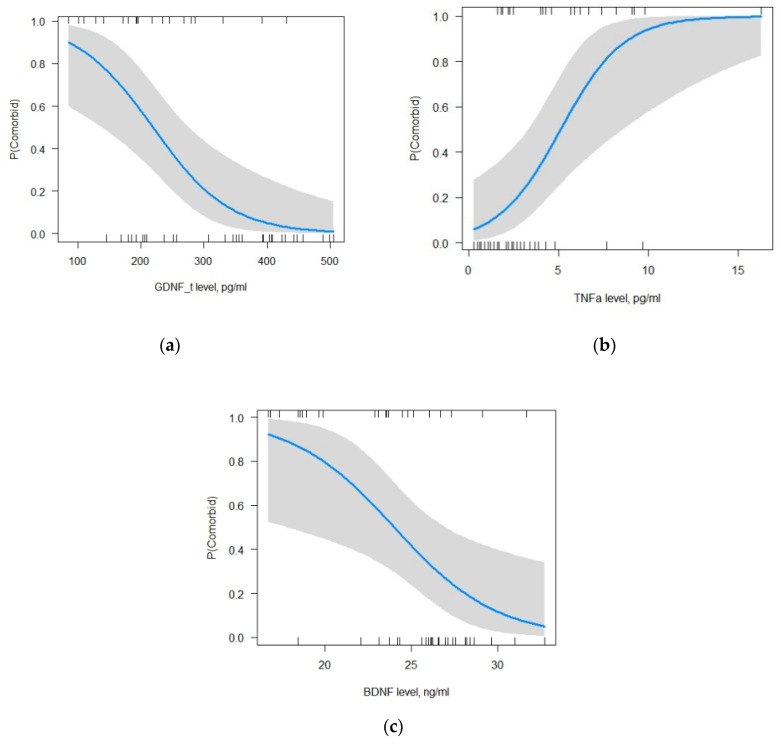
Dependence of changes in GDNF in LF (**a**), TNF-α (**b**), and BDNF (**c**) levels on the likelihood of developing comorbid depression and epilepsy. Vertical lines along the x-axis indicate observations; a gray bar along the blue line indicates a 95% confidence interval.

For FE + MDD, the multiple logistic regression method showed that out of the seven tested variables, GDNF in LF and BDNF and TNF-α in BS had a statistically significant effect on the development of FE + MDD, *p* < 0.05: GDNF in LF (β0 = 14.09, β1 = −0.01, *p* = 0.01; ORβ0 = 1.32*106 (95%CI: 421.14–3.31*1011), ORβ1 = 0.99 (95%CI: 0.97–0.99)); BDNF (β0 = 14.09, β2 = −0.53, *p* = 0.003; ORβ0 = 1.32*106 (95%CI: 421.14–3.31*1011), ORβ2 = 0.59 (95%CI: 0.38–0.79)); and TNF-α (β0 = 14.09, β3 = 0.57, *p* = 0.004; ORβ0 = 1.32*106 (95%CI: 421.14–3.31*1011), ORβ3 = 1.77 (95%CI: 1.27–2.84)). The increase in the levels of GDNF in LF and BDNF as well as a decrease in TNF-α levels reduce the likelihood of developing FE + MDD (Figure 5a–c). 

According to the selected model, reducing the GDNF level in LF by one unit increases the probability of developing FE + MDD by 2%, reducing the BDNF level in BS by one unit elevates the probability of developing FE+ MDD by 41%, and increasing the TNF-α level by one unit elevates the probability of developing FE+ MDD by 77% (Change in Odds%: (OR-1)*100). Test for multicollinearity showed that the values of all predictors were close to one. It means that there was no correlation between the explanatory variables of the model (GDNF in LF = 1.34, BDNF = 1.5, TNF- = 1.15). The AUC of the selected model was 0.94 (accuracy 0.92, precision 0.9, sensitivity 1, f1-score 0.95). Pseudo R-squared values indicate that the model explains 57.3–72.9% of the data (Pseudo.R.squared/McFadden = 0.57, Cox and Snell = 0.54, Nagelkerke (Cragg and Uhler) = 0.73). The likelihood ratio test is 41.57 with a *p*-value of less than 0.05 that points to the good predictive power of the selected model. After compiling a classification table with the test data of 13 participants (20% of the entire sample) obtained from this model with a cut-off point of 0.5, the precision of the model is found to be 90%, the recall is found to be 100%, and the F1 score of the model is found to be 94.7%. The accuracy of the selected model, with a cut-off point of 0.5 for the binary classification, is 92.3% (a cut-off point of 0.75 is required for 92.3% accuracy).

## 3. Discussion

### 3.1. Neurotrophic Factors in Epilepsy 

Several lines of evidence suggest that neurotrophic factors are highly involved in the development of acquired epileptic syndromes, though they can have contrasting effects [19]. BDNF is the most widely distributed neurotrophin in the central nervous system. BDNF and its receptor, tropomyosin-related kinase receptor type B (TrkB), play an active role in the numerous areas of the adult brain, where they regulate the neuronal activity, function, and survival. The upregulation and downregulation of the BDNF expression are critical for the physiology of neuronal circuits and brain functioning [20,21]. BDNF is used in the developmental assessment, treatment monitoring, and pharmacotherapy of selected diseases, in particular epilepsy and depression, though two controversial views still exist that BDNF inhibits or promotes epileptogenesis [22].

However, the neurotrophic and neuroprotective properties of BDNF can be potentially used to treat epilepsy, e.g., by inhibiting BDNF-TrkB signaling and reinforcing the NPY system [6,23]. 

Though not yet deeply explored, the therapeutic potential of GDNF for hippocampus-related neurological disorders (including epilepsy) is regarded as fairly high [17]. The involvement of GDNF in the pathogenesis of epilepsy has been studied using animal models. In particular, in the rat models of epilepsy, GDNF delivered by various routes exhibited a beneficial effect by suppressing seizures and/or reducing their frequency [11,24,25]. Therefore, it is more surprising that the information on the GDNF levels in BS or plasma of FE is scarce.

In the present study, GDNF was assessed in BS, and it did not differ in FE, MDD, and FE + MDD compared to the healthy controls. This corresponds to the results of the single study related to the comparison of the GDNF content in blood plasma of healthy individuals and patients with epilepsy (including focal and generalized epilepsies): no difference between patients with epilepsy and controls has been found [26]. Importantly, in our study, GDNF in LF was significantly reduced in FE, FE + MDD, and MDD. Our recent study found that the changes in GDNF in LF of patients with bipolar disorder and MDD [16] were similar to those found in FE and in FE + MDD in the present study. However, in the present study, depression did not influence the GDNF content either in LF or BS. It can be suggested that FE + MDD did not show a much lower GDNF level in LF because the potential for a further decrease was exhausted. Thus, a reduction in the GDNF level in LF might be one of potential biomarkers of both depression and epilepsy.

Other neurotrophic factors show different alterations in LF of epilepsy patients. We showed BDNF decrease both in BS and LF of epilepsy patients [15], while, on the contrary, CNTF in these media was increased [14]. In the present study, serum BDNF was reduced in all groups of patients compared with HC. Overall, these data confirm the results of other studies that suggest an involvement of BDNF in the pathogenesis of epilepsy [6] and depression [27] and those of our previous study [28]. 

An interesting finding is a significant increase in serum GDNF and BDNF in FE taking valproates. This is in line with several studies, which showed that valproates significantly increase GDNF and BDNF expression in rat C6 glioma cells [29] and astrocytes [30,31]. Similarly, we have previously shown that FE receiving valproates as mono- or polytherapy had higher BDNF level in BS [15]. McGonigal et al. [32] also found the effects of valproates on serum BDNF levels in patients with epilepsy. The effects of valproates are known to be mediated by epigenetic mechanisms, including histone deacetylases, and the modulation of BDNF and GDNF by valproates is pivotal to orient neurons toward a neuroprotective status and promote the organization of dendritic spines [33]. 

### 3.2. Relationship between Neurotrophic Factors, HPA Axis, and Inflammation

The HPA axis as well as inflammation are involved in the pathophysiology of many neurological and neuropsychiatric disorders. Glucocorticoid hormones ensure the coordinated functioning of crucial mechanisms of hippocampal plasticity: neurogenesis, glutamatergic neurotransmission, microglia and astrocytes, the systems of neurotrophic factors, neuroinflammation, etc. [5]. Regulatory mechanisms are miscellaneous and include the direct action of glucocorticoids through their receptors and the effects of HPA axis on numerous interactions between various systems and components. 

The results of many clinical and animal studies confirm that disturbed neurotrophic factor systems, especially BDNF, and inflammation are two important risk factors in the pathogenesis of depression [34,35]. The elevated levels of inflammatory mediators may reduce the expression of BDNF, while BDNF plays a negative regulatory role in neuroinflammation. TNF-α is one of the most extensively investigated mediators in the studies on inflammatory factors in human epilepsy [36] and depression [37]. In the present study, the concentrations of TNF-α in BS of all patient groups were augmented compared to HC, and this was accompanied by a significant decrease in the GDNF levels in LF and the BDNF levels in BS. These data confirm the concept that impaired immunoregulatory mechanisms may induce systemic neuroinflammation and the decrease in trophic support. 

In the present study, higher basal cortisol levels were found in FE + MDD and MDD compared to those in HC, confirming many previous reports. No significant increase was found in the cortisol level in FE. Cano-López and González-Bono have analyzed the data of 38 studies on cortisol levels in adults with epilepsy and found higher basal cortisol levels in PWE in only 45% of studies compared with their respective controls [38]. Taking into consideration that epilepsy may be regarded as a model of chronic stress [4], the lack of pronounced cortisol activation in FE may be due to the deeper exhaustion of HPA axis in some patients with epilepsy. In our previous study, it was shown that, in MDD, serum GDNF and cortisol concentrations were significantly higher than those in FE [28]. When such groups of patients were compared in the present study, the cortisol level in the serum of the MDD group was insignificantly higher than that in FE, though showing a statistically significant trend (*p* < 0.09). However, similar to the previous data, the changes in levels of GDNF in LF and BDNF and cortisol in BS, assessed in the present study, did not depend on the etiology of epilepsy and were related to epilepsy in general, independent of its etiology [28].

An increasing number of studies support the hypothesis that neuropsychiatric disorders, including epilepsy and depression, are associated with cell-mediated systemic inflammation. Changes in blood cellular ratios, on the one hand, may be due to disease, and on the other hand, due to the effect of the drugs taken. Our data showed that white blood cell count and platelet-to-neutrophil ratio were reduced in FE compared to HC, and this may be a consequence of a more pronounced/long-lasting effect of valproates [39]. A higher percentage of monocytes in FE may indicate a more pronounced monocyte activation in epilepsy compared to other groups [40]. FE + MDD and MDD showed augmented levels of lymphocytes (%), presumably due to a higher percentage of patients taking antidepressants in the MDD group [41]. MDD had higher prolactin levels compared to HC, most likely this is related to a higher percentage of patients taking antipsychotics in the MDD group [18]. This is a usual adverse effect of many antipsychotic drugs and antidepressants. The highest degrees of hyperprolactinemia are associated with taking amisulpride, risperidone, and paliperidone, or adjunctive antipsychotic treatment; therapy with selective serotonin reuptake inhibitors (escitalopram, paroxetine, or sertraline) can induce hyperprolactinemia in patients with depression.

To find a combination of biochemical parameters capable of predicting the probability of developing FE, MDD, and their comorbid state, we have created three models using the logistic regression method. To generate the models, we used the cortisol level, representing the functioning of HPA axis, TNF-α level, reflecting the activation of inflammatory processes, and the concentrations of neurotrophic factors (GDNF in LF and BDNF in BS), reasonably assuming the involvement of all these systems in the pathophysiology of both epilepsy and depression. We used some sociodemographic characteristics, in particular the age and gender of participants, to ensure the absence of their influence on the above diseases. We have shown that the probability of developing FE can be estimated with a moderate predictive power using GDNF in LF and BDNF in BS; the probability of developing MDD can be estimated with a high predictive power using GDNF in LF, cortisol in BS, and TNF-α in BS, while the probability of developing FE with MDD can be estimated with a high predictive power using GDNF in LF, TNF-α in BS, and BDNF in BS. Comparing the combinations of biochemical predictors for FE, MDD, and their comorbid state and forecasting the likelihood of their development suggest that pathophysiological processes in FE, MDD, and FE + MDD include both identical and specific features. Thus, in the predictive model of FE, neurotrophic factors played an important role, with a greater degree of reduction observed in BDNF levels. Simultaneously, in the model predicting MDD, the most important input exhibited an increase in the level of TNF-α. Finally, in the model predicting FE with MDD, the maximal contribution was made by a decrease in the level of BDNF and an increase in the level of TNF-α. 

## 4. Materials and Methods

### 4.1. Subjects

A group of 60 consecutive patients over 18 years old diagnosed with focal epilepsy (FE, *n* = 32) and with focal epilepsy and comorbid MDD (FE + MDD, *n* = 28) and a comparison group of patients of similar age and gender proportion with MDD (MDD, *n* = 46) were recruited at the Moscow Research and Clinical Center for Neuropsychiatry between October 2020 to August 2021. Furthermore, 39 generally healthy volunteers of similar age and gender without any signs of mental disorder, both at the time of including into the study and as per their medical records, were enrolled as healthy controls. 

Inclusion criterion for the group with epilepsy was the presence of focal epilepsy, thoroughly diagnosed through the consensus of at least two experienced neurologists according to the criteria for epilepsy, based upon the International League Against Epilepsy (ILAE) classification [42,43]. All FE underwent electroencephalography (EEG) and magnetic resonance imaging (MRI) of the brain. Subjects were excluded from the study if they had no records of seizure frequency, generalized, combined, or epilepsy of unknown origin, significant psychiatric comorbidity (excluding depression), history of psychogenic nonepileptic seizures, presence of serious somatic, neurological, or systemic disorders. All patients were examined by an experienced psychiatrist to diagnose depression and exclude other psychiatric comorbidities. 

Inclusion criteria for the group with MDD were the diagnosis of current depressive episode, age of 18 years and above, and the ability to provide an informed consent and comply with the study protocol. The exclusion criteria were cognitive impairment (score of less than or equal to 24 on the Mini-Mental State Examination (MMSE) [44], current or past psychotic disorders, alcohol or substance use disorders, manic/hypomanic symptoms/episodes, severe concomitant somatic (e.g., diabetes mellitus and autoimmune or oncological diseases), or neurological (e.g., Alzheimer’s and Parkinson’s diseases) disorders. People with initial or mild manifestations of somatic diseases, such as essential hypertension, ischemic heart disease, or cardiac arrhythmias, were not excluded. A mental disorder diagnosis was established by a psychiatrist using a Mini-International Neuropsychiatric Interview (MINI v 7.0.2). 

The patients were not treatment naïve and received appropriate medications (treatment as usual) prescribed by an experienced psychiatrist. The pharmacotherapy of patients with epilepsy, in addition to antipsychotics, antidepressants, tranquilizers, and valproates, included sodium channel blockers (eslicarbazepine, fenitoin, lamotrigine, lacosamide, oxcarbazepine, ocarbamazepine), GABA inhibitors (benzobarbital, diazepam, fenazepam, phenobarbital, clonazepam), neurotransmitter release inhibitors (pregabaline, gabapentine, levetiracetame, brivaracetame, ethosuximide), zonisamide, and topiramate.

The Russian version of the Beck depression inventory-II (BDI-II) was used to evaluate the severity of depression [45]. 

All patients signed an informed consent form prior to participating in the study. This study adhered to the tenets of the Declaration of Helsinki and had the approval of the local ethics committee (#42, 23.08.2019) with informed consent forms obtained from all subjects.

### 4.2. Samples

Biochemical and hormonal indices were measured in blood serum obtained from fasting morning venous blood. Samples were collected in Gel/Clotting activator S-Monovette tubes and centrifuged at 2000× *g* for 10 min at 8 °C on an Allegra X-30R Centrifuge (Beckman Coulter, Brea, CA, USA). 

Stimulated LF (secreted by the lacrimal gland in response to a mechanical stimulation of the cornea) was sampled using a pipette at a volume of 100–200 μL from the lower conjunctival fornix of one randomly selected eye. Samples were stored at −80 °C in polypropylene tubes (Sarstedt GmbH, Nümbrecht, Germany) and analyzed within 3 months from sampling. Upon thawing, samples were centrifuged at 4000× *g* for 15 min at 4 °C to ensure a complete removal of debris. Based upon the previously described methods [46], an acid treatment procedure was implemented to allow the quantification of total GDNF levels in biological samples. 

### 4.3. Assessment of Biochemical Indices and Hormones

Concentrations of GDNF were measured in biological fluids using Human GDNF ELISA Kit (Ray Biotech, Norcross, GA, USA), according to the manufacturer’s instructions. The sensitivity of the assay (minimum quantifiable value) was 4.0 pg/mL. All measured values were in the validated assay range. If sample volume permitted, two replicates were used. The concentrations of brain-derived neurotrophic factor (BDNF) were determined with enzyme-linked immunosorbent assay (ELISA) in blood serum using corresponding Quantikine ELISA test systems (R&D Systems, Minneapolis, MN, USA). Cortisol and thyroid-stimulating hormone (TSH) were measured in blood serum via competitive enzyme immunoassay using applicable kits (Beckman Coulter, Brea, CA, USA) and an ACCESS^®®^ 2 immunoassay system (Beckman Coulter, USA). The concentration of tumor necrosis factor-α (TNF-α) was determined using ELISA with corresponding human high sensitivity ELISA kits (eBioscience, Bender MedSystems GmbH, Vienna, Austria). Adrenocorticotropic hormone (ACTH) was assessed using enzyme immunoassay kits from Biomerica (Irvine, CA, USA). GDNF, BDNF, TNF-α, and ACTH levels were measured on an automated enzyme immunoassay analyzer (ChemWell 2910, Awareness Technologies Inc., Palm City, FL, USA). Routine biochemical parameters and ions were determined in blood serum with a biochemical automated analyzer, Beckman Coulter AU 680 (Beckman Coulter, Brea, CA, USA), using corresponding kits (Beckman Coulter, Brea, CA, USA). Complete blood count with differential white blood cell count (CBC with diff) and hemogram were performed on an automated analyzer LH-500 (Beckman Coulter, Brea, CA, USA). 

### 4.4. Statistical Analysis 

Statistical analysis was performed using STATISTICA 10.0 (StatSoft Inc., Tulsa, OK, USA) and GraphPad Prism version 9.4.1. software (GraphPad Software, Inc., San Diego, CA, USA) and in the R programming environment on the RStudio version 2023.06 platform. 2 (2009–2023, Posit Software, PBC) using the following libraries: ggplot2, ROCR, dplyr, tidyr, MASS, caret, and margins. The normality of distribution was determined using the Shapiro–Wilk and Kolmogorov–Smirnov tests. Fisher’s exact test was used to compare qualitative data. To compare quantitative data between several unrelated groups depending on their distribution, either the ANOVA test with post hoc analysis using Tukey’s test or the Kruskal–Wallis test with post hoc analysis using Dunn’s test were applied. Correlation analysis was carried out using the Spearman rank correlation test. The data in the graphs and in the tables are presented as mean with SD, median with interquartile range, or percents. Differences were considered significant at *p* < 0.05. A backward logistic regression model was used. The significance level for each variable’s entry to the model was set at 0.05. 

## 5. Conclusions

Our results reveal a high value for the assessed levels of lacrimal GDNF as a non-invasive biomarker in FE, MDD, and FE with MDD in contrast to serum GDNF levels, which were not different between healthy controls and the various patient groups. This result supports the use of LF as a promising source of disease biomarkers [47], with LF analysis being a way for opening a window into the brain. Epilepsy as a stress-associated disorder shares many vital links of depression pathogenesis, HPA axis disturbances, inflammatory alterations, and trophic support decrease [5]. Models obtained using logistic regression in this study suggest that changes in these systems in FE, MDD, and FE + MDD include both similar processes, potentially important for comorbidity, and specific mechanisms for either epilepsy or depression. The results obtained confirm the involvement of the HPA axis, the system of neurotrophic factors (GDNF, BDNF), and inflammation (TNF-α) in the pathogenesis of epilepsy and depression. The models created in this study can be further developed and used both to predict the course of emotional disorders in patients with epilepsy and to form the basis of personalized approaches used in their therapy.

## Figures and Tables

**Table 1:** Characteristics of patients and healthy control group.

Parameter/ Group	HC M ± SD/M [Q1; Q3] (*n* = 39)	FE M ± SD/M [Q1; Q3] (*n* = 32)	FE + MDD M ± SD/M [Q1; Q3] (*n* = 28)	MDD M ± SD/M [Q1; Q3] (*n* = 46)
Age, years	34.6 ± 10.6	37.4 ± 12.2	37.4 ± 10.6	36.7 ± 7.6
Gender (male/female), %	23/77	41/59	25/75	24/76
Age, years (male/female)	30.0 ± 4.6/35.6 ± 11.7	36.9 ± 12.6/37.7 ± 11.6	35.0 ± 6.2/37.9 ± 11.4	34.7 ± 7.7/37.3 ± 7.5
Education(sec./higher), %	49/51	44/56	68/32	56/44
Employment (−/+), %	13/87	59/41	52/48	54/46
MMSE	-	29 [28;30]	27 [26;29]	29 [28;30]
Beck II	-	7 [3;13]	28.5 [20;32] ^#^	30.5 [26;34] **
Epilepsy duration, years	-	16.9 ± 9.8	16.1 ± 10.7	-
Take antipsychotics, %	-	41	73	97
Take antidepressants, %	-	75	95	99
Take tranquilizers, %	-	53	64	68
Take sodium channel blockers, %	-	72	77	11
Take GABA inhibitors, %	-	0	9	0
Take neurotransmitter release inhibitors, %	-	41	36	0
Take valproates, %	-	34	32	5
Take AMPA receptor antagonists, %	-	16	5	0
Take other (zonisamide, topiramate), %	-	16	18	0
Focal Onset Aware Seizure, %	-	41	55	-
Focal Onset Impaired Awareness, %	-	72	64	-
Focal to bilateral tonic–clonic seizure, %	-	72	86	-
Frequency of seizures: absence during the year/<1 per month/1–3 per month/1 or more seizures per week, %	-	25/38/16/19	14/27/18/41	-
Platelets, PLT, 10^3^/µL	250.0 ± 57.1	245.2 ± 69.5	266.1 ± 64.6	239.1 ± 58.0
Erythrocytes, RBC, 10^6^/µL	4.7 ± 0.5	4.7 ± 0.4	4.5 ± 0.5	4.6 ± 0.5
Hemoglobin, Hb, g/L	138.4 ± 13.5	138.3 ± 14.2	138.2 ± 13.9	138.7 ± 13.8
White blood cell, WBC, 10^3^/µL	6.6 ± 1.7	5.6 ± 1.5 *	6.4 ± 1.8	6,3 ± 1.6
Neutrophils, NE, %	58.0 ± 7.2	54.5 ± 9.1	54.2 ± 8.4	52.1 ± 10.0 *
Lymphocytes, LY, %	31.2 ± 6.8	33.6 ± 9.0	35.1 ± 7.9 *	36.6 ± 9.3 *
Monocytes, MO, %	7.5 [6;8.6]	8.6 [7.3;10.3] *	7.1 [6.2;9.5]	7.8 ± 1.8
Eosinophils, EO, %	2 [1;2.6]	2 [1.2;3.1]	1.9 [1.6;2.7]	2.6 [1.5;3.9]
Basophils, BA, %	0.7 [0.5;1]	0.6 [0.4;1.1]	0.7 [0.45;0.9]	0.9 [0.6;1.2]
NE/LY, NLR	1.73 [2;1.5]	1.7 [1.2;2.3]	1.6 [1.1;2.1]	1.4 [1.1;2]
LY/MO, LMR	4.2 [3.6;5]	3.9 [2.9;4.6]	4.4 [3.5;6]	4.7 [3.8;6]
PLT/NE, PNR	68.66 [54.4;78.3]	84.7 [69.2;111.4] *	66.1 [49.3;89.6]	78.4 [57.5;94.5]
PLT/LY, PLR	123.7 [104.7;155.2]	131.1 [101.1;161.2]	103 [79;136]	103.2 [87;138]
PLT/MO, PMR	616 [448;639]	551 [415.2;630.1]	474 [383;589]	505.5 [411;628]
Total bilirubin, µmol/L	8.85 [6.6;12.1]	9.7 [7.2;12.3]	7.6 [6.6;10.2]	10.7 [7.5;15.5]
Glucose, mmol/L	4.95 [4.5;5.4]	5.1 [4.8;5.5]	5.1 [5;5.5]	4.9 [4.6;5.2]
Creatinine, µmol/L	78 [69;86]	85 [75.5;93.5]	79 [74;89]	82 [73;92]
Urea, mmol/L	4.3 [3.4;5.2]	3.5 [3.1;5]	3.9 [3.3;5]	3.8 [3.4;4.5]
Cholesterol, mmol/L	5.2 ± 1.2	6.0 ± 1.3	5.3 ± 1.3	4.2 ± 1.2
Triglycerides, mmol/L	1.4 ± 0.5	1.5 ± 0.8	1.3 ± 0.6	1.7 ± 0.6
K, mmol/L	4.3 ± 0.4	4.3 ± 0.5	4.4 ± 0.4	4.5 ± 0.4
Na, mmol/L	136.0 ± 4.0	140.5 ± 3.1	140.0 ± 4.7	139.4 ± 3.4
Ca, mmol/L	1.2 ± 0.1	1.3 ± 0.4	1.4 ± 0.5	1.2 ± 0.2
TSH, ulU/mL	1.7 [1.26;2.27]	1.8 [1.3;2.6]	2.2 [1.6;2.8]	2 [1.3;2.8]
ACTH, pg/mL	9.6 [7.9;15.7]	10.1 [8;13]	11.6 [7.5;15.7]	12.5 [10.3;14.7]
Prolactin, ng/mL	11.17 [8.2;15]	11.7 [8.7;19.2]	12 [9.3;16.7]	23.3 [13.5;39.2] ∗
GDNF_t, pg/mL	355.2 [209.5;426.5]	216.7 [157.5;247.5] *	194.5 [151.8;263.4] *	188.8 [131.5;227.5] *
GDNF_s, pg/mL	158.3 [130.2;200.2]	135.8 [115.5;160.8]	146.1 [106.7;195.3]	143.4 [128.6;186.6]
BDNF, ng/mL	25.83 ± 2.74	23.12 ± 4 *	22.61 ± 3.98 *	23.25 ± 4.37 *
Cortisol, nmol/L	295 [226;387]	363 [264.2;478.3]	409 [302.3;489] *	426 [358;514.1] *
TNF-α, pg/mL	1.8 [0.9;2.9]	3.3 [2.3;4.8] *	4.6 [2.5;6.7] *	4.95 [3.78;6.43] *

Depending on the distribution, the data are presented as mean ± SD (M ± SD) for normal distribution or median with interquartile range (M [Q1; Q3]). Statistical significance: * *p* < 0.05, ** *p* < 0.01, compared to HC; ^#^ *p* < 0.05, compared to FE. Differences between groups with quantitative data were assessed using one-way ANOVA (with post hoc Tukey test) or Kruskal–Wallis test (with post hoc Dunn test). For qualitative data, Fisher’s exact test was used. MMSE—Mini-Mental-State Examination, BDI-II—depression inventory–II, TSH—thyroid-stimulating hormone, ACTH—adrenocorticotropic hormone, GDNF—glial cell line-derived neurotrophic factor, BDNF—brain-derived neurotrophic factor, and TNF-α—tumor necrosis factor α.

**Table 2:** Correlations of GDNF level in LF, GDNF, BDNF, cortisol, and TNF-α levels in BS with age.

	GDNF in LF	GDNF in BS	BDNF	Cortisol	TNF-α
N (number of observations)	145	145	144	144	131
R (Spearman)	−0.12	−0.01	0.03	0.11	0.16
*p*-value	0.17	0.88	0.69	0.18	0.1

**Table 3:** The nosological structure of patients with focal epilepsies.

	SFE (Multiple Cause) M ± SD/M [Q1; Q3] (*n* = 19)	SFE (after Epi Surgery) M ± SD/M [Q1; Q3] (*n* = 8)	SFE (Traumatic Brain Injury, Stroke, Cerebrovascular Disorders) M ± SD/M [Q1; Q3] (*n* = 13)	CFE (Not Established) M ± SD/M [Q1; Q3] (*n* = 20)
Age, years	36.9 ± 11.4	34.9 ± 9.0	40.4 ± 14.1	41.9 ± 11.4
Gender (male/female), %	37/63	25/75	31/69	30/70
Education(secondary/higher), %	47/53	62/38	46/54	65/35
Employment (−/+), %	43/57	62/38	62/38	61/39
MMSE	28 [26;29]	30 [26;30]	26 [25.5;28]	29 [26;30]
Beck II	13 [6;29] **	4.5 [2;16]	21 [13;2] **	16 [10;25] **
With MDD, %	37 **	0	69 **	43 **
Cortisol, nmol/L	399 [300;460.5]	337 [306.5;454.1]	391 [366;461]	411 [275.3;505.1]
TNF-α, pg/mL	3 [2.1;4.45]	3.8 [2.8;6]	5.9 [3.95;8] *	4.3 [2.52;5.9]
GDNF, pg/mL in LF	156.8 ± 57.4	141.0 ± 38.3	149.2 ± 41.0	131.5 ± 33.5
GDNF, pg/mL in BS	209.0 ± 73.8	191.3 ± 47.8	213.6 ± 79.0	213.7 ± 79.7
BDNF, ng/mL	19.9 [18.5;24.3]	24.3 [20.4;25.1]	24.5 [18.9;26.2]	22.67 [19.4;26.6]

Depending on the distribution, the data are presented as mean ± SD (M ± SD) or median with interquartile range (M [Q1; Q3]). Statistical significance (*p* < 0.05): * compared to SFE (multiple cause) and ** compared to SFE (after epi surgery). Differences between groups with quantitative data were assessed using one-way ANOVA (with post hoc Tukey test) or Kruskal–Wallis test (with post hoc Dunn’s test). For qualitative data, Fisher exact test was used. SFE—symptomatic focal epilepsy; CFE—cryptogenic focal epilepsy.

## Data Availability

The data generated in the present study are available upon a reasonable request.
